# Achieving Pregnancy Using Primary Care Interventions to Identify the Fertile Window

**DOI:** 10.3389/fmed.2017.00250

**Published:** 2018-01-09

**Authors:** Thomas P. Bouchard, Richard J. Fehring, Mary M. Schneider

**Affiliations:** ^1^University of Calgary, Calgary, AB, Canada; ^2^College of Nursing, Marquette University, Milwaukee, WI, United States

**Keywords:** natural family planning, fertility awareness, family planning, subfertility

## Abstract

**Objective:**

To determine the effectiveness of achieving pregnancy with focused intercourse in the fertile window identified using natural fertility indicators.

**Methods:**

24-cycle prospective effectiveness study.

**Setting:**

A North American web-based fertility monitoring service.

**Participants:**

256 North American women aged 20–43 (mean age 29.2 years) seeking to achieve pregnancy.

**Intervention:**

Participants identified their fertile window with either electronic hormonal fertility monitoring or cervical mucus monitoring, or both, and recorded their observations on an online fertility tracking system.

**Main outcome measures:**

Pregnancies were validated by nurses with an online self-assessed pregnancy evaluation form. Survival analysis was used to determine pregnancy rates.

**Results:**

There were 150 pregnancies among the 256 participants with an overall pregnancy rate of 78 per 100 women over 12 menstrual cycles. There were 54 pregnancies (68%) among the 80 women using the fertility monitor, 11 pregnancies (46%) among the 24 women using mucus monitoring, and 90 (63%) among the 143 women using both mucus and monitor. The 12-cycle pregnancy rates per 100 women were 83 (monitor group), 72 (mucus group), and 75 (mucus and monitor group). Pregnancy rates reached 100% at 24 cycles of use for those women using the hormonal fertility monitor.

**Conclusion:**

Use of the hormonal fertility monitor alone seems to offer the best natural estimate of the fertile phase of the menstrual cycle for women wishing to achieve a pregnancy. Focusing intercourse through 24 menstrual cycles can be beneficial for achieving pregnancy.

## Key Points

- Instead of having a waiting period, primary care providers can begin by addressing common primary care concerns to optimize fertility naturally (1) (see Table 1).
- One tool that primary care providers can recommend is the use of a hormonal fertility monitor for 12–24 months to assist couples to achieve pregnancy by focusing intercourse in the fertile window.

**Table 1:** Some examples of primary care-based recommendations for optimizing natural fertility.

Lifestyle recommendations (1)	Cycle-based recommendations	Medical management
Harvard fertility diet (2) Smoking cessation (3, 4) Caffeine and alcohol reduction (5, 6) Use of multivitamins (7, 8)	Identifying ovulation and focusing intercourse (current study) Identifying luteal phase deficiency (9)	Diagnosing and treating polycystic ovarian syndrome (10) Diagnosing and treating thyroid disorders (11)

## Introduction

A common approach in primary care to deal with couples seeking advice on achieving pregnancy is to encourage regular sexual intercourse, and to return after a year of trying for an infertility work-up which often leads to the recommendation of using Artificial Reproductive Technologies (ARTs) (12, 13). The expectant approach could be argued based on estimates that 85 per 100 women who have unprotected intercourse over 12 months would conceive according to Trussell (14). Others have even suggested that current treatments, focusing mainly on ARTs, are no better than expectant management (15, 16), so couples should not be offered any interventions in the first 12 months of trying aside from the recommendation of frequent intercourse (17). However, many couples are looking for ways to optimize fertility rather than an “expectant” approach. Instead of having a waiting period, primary care providers can begin by addressing some common primary care concerns to optimize fertility naturally (1) (see Table 1). While there are other options for natural fertility evaluation and treatment that require specialized training (18), the approaches summarized in Table 1 can begin as soon as couples are trying to achieve pregnancy (and need not wait for a full year), and all of them can be initiated by a primary care provider without significant specialized training. The current study specifically addresses the identification of the fertile window to predict the most fertile time of the cycle.

A recent study of a cohort of women trying to achieve pregnancy determined that the main reason for lack of results was due to mistiming of intercourse, i.e., having intercourse outside of the fertile phase of the menstrual cycle (19). Other studies have consistently demonstrated, even among sub-fertile couples trying to achieve pregnancy, that many (up to 75%) have little knowledge of the actual biological window of fertility (20). While it makes sense to focus intercourse on the most fertile days of the menstrual cycle, the evidence for this intervention to date is weak and the debate continues as to whether frequent intercourse is just as effective and less stressful (21).

Several studies have demonstrated that the window of fertility during the menstrual cycle includes the day of ovulation and the 5 days prior (22, 23). This fertile window is based on sperm survival of up to 5 days in good quality cervical mucus and egg survival of up to 24 h. Other studies have also shown that the most fertile days for achieving pregnancy are the 2–3 days prior to the day of ovulation (24). Therefore, it makes sense to focus intercourse in this fertile window to increase the chance of pregnancy. However, having frequent intercourse might work as well, since some of those frequent acts of intercourse may land on a day in the fertile window. A recent Cochrane systematic review of randomized controlled trials for timing intercourse with and without ovulation prediction suggested that there was insufficient data to conclude the efficacy of the intervention, mainly because of low quality evidence in the studies that have been done to date (25).

Some prospective studies have shown increased probabilities of pregnancy when couples use focused intercourse with the aid of readily observable physiologic signs designed to estimate the biological fertile window (26, 27), including focusing intercourse on days of good quality cervical mucus (28–30), and combining cervical mucus with body temperature measurements (31). A more recent study randomized participants to cervical mucus monitoring versus frequent intercourse two to three times per week and found no increased probability of pregnancy in the mucus monitoring group (32).

Aside from mucus and temperature observations, the fertile window can also be identified with the use of electronic hormonal fertility monitors. One such device, the ClearBlue Fertility Monitor (CBFM, Swiss Precision Diagnostics, Geneva, Switzerland), detects estrogen and luteinizing hormone metabolites in the urine, and provides the user with a daily indication of “Low,” “High,” and “Peak” fertility (33). Using the CBFM, a recent study randomized 1,000 women volunteers into 2 groups; half received a hormonal fertility monitor and the other half were a control group who were asked to do what they wished to achieve a pregnancy (19). The pregnancy rate was statistically higher for the fertility monitor at 22.7% compared with the control group at 14.4% (*p* = 0.006). This study was limited as its duration was only two menstrual cycles in length, participants were not sub-fertile, and control group participants may have used other methods of estimating the fertile phase of the menstrual cycle. A more recent prospective randomized controlled trial used a digital urinary ovulation LH predictor kit and compared this to a group of women who were instructed to have frequent intercourse (every 2–3 days) without using any self-observation fertility indicators, and they found that the women using the digital LH test had higher pregnancy rates (43%) than the control group (30%) (34).

In another study using the CBFM, Mu and Fehring (35) compared pregnancy rates for couples having intercourse on a “High” or “Peak” day compared to a “Low” day within the estimated fertile window. When couples focused on the “High” or “Peak” days of the estimated fertile window, the pregnancy rate was 87 per 100 women over 12 months compared to only 5 per 100 women for couples who used only the “Low” days in the estimated fertile window. This study demonstrated that a large proportion of couples trying to conceive may benefit from focused intercourse with the use of the CBFM for identifying the fertile window of the menstrual cycle.

While the Mu and Fehring (35) study compared intercourse only on ‘High’ and ‘Peak’ days to intercourse only on ‘Low’ days, the aims of the current study were to determine and compare the 12 and 24-cycle extended effectiveness rates of achieving pregnancy for women who indicated their intention to achieve pregnancy (intention to treat) using an intervention that can be taught by primary care providers (the use of the CBFM or cervical mucus monitoring or both) with focused intercourse during the self-estimated fertile window of the menstrual cycle.

## Materials and Methods

### Participants

North American women were recruited from April of 2008 through April of 2015 by an announcement of a new fertility monitoring web site in an online fertility discussion forum for health professionals and by word of mouth online. The main criterion to be in this study was that the female participant indicated the intention to achieve pregnancy and had at least one menstrual cycle of charting (learning the method can be either in person with a trained Marquette Method professional nurse or physician, or *via* online instruction). At the time of registration on the website, participants were asked to sign an online consent form that requested they use the site for charting and provide feedback to the developers. There were 256 participants who met the criteria for this study. Women were not asked if they had a history of fertility problems, but we included both those with proven fertility (who already had children) and those who had not been pregnant. The use of the Marquette Method online system for statistical analysis has been approved by the Marquette University Office of Research Compliance (HR-1597).

### Online Fertility Tracking System

The fertility health web site (https://nfp.marquette.edu) provides information on fertility health, short instructional videos, downloadable menstrual cycle charts, instructions on how to observe and record natural indicators of fertility, and instructions for achieving and avoiding pregnancy. Women who register on the web site have access to the discussion forums and consultation from professional nurses and physicians who have expertise in the use of fertility monitoring, as well as a bioethicist. The nurses periodically update the web site with research on fertility, including how to optimize fertility.

**Figure 1:**
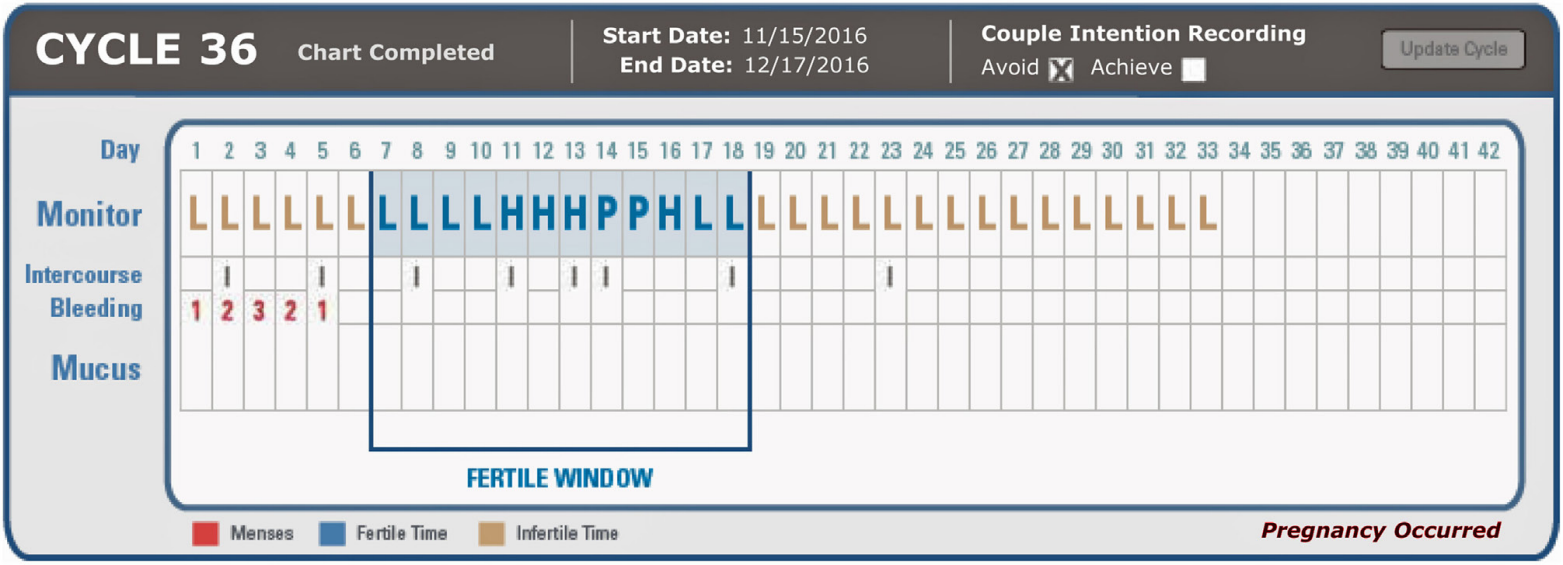
Example of online charting system indicating ClearBlue Fertility Monitor results (L/H/P for Low, High, Peak, respectively), intercourse frequency, bleeding (1 = light, 2 = moderate, 3 = heavy), and mucus findings (not recorded in this participant).

The online charting system (Figure 1) has sections for recording the results of both the CBFM and cervical mucus. The charting system illustrates the three fertility levels (L = Low, H = High, or P = Peak fertility) of the fertility monitor or cervical mucus observations. Menses can be recorded on a scale of 1–3 (1 = light, 2 = moderate, and 3 = heavy menstrual flow) and intercourse can also be recorded (“I”). The charting system requires that the user indicate intention of use (to achieve or avoid pregnancy) at the beginning of each cycle. The charting system automatically indicates (in light blue) the fertile phase as the user charts (see Figure 1). Participants can use the charting system with either the CBFM or cervical mucus monitoring or both indicators. The online system automatically calculates the estimated fertile window based on the built in algorithm (36). The CBFM detects a rising level of urinary estrogen when indicating a “High” fertility level and a threshold level of urinary luteinizing hormone when indicating a “Peak” recording. Users of the CBFM tested the first morning concentrated urine with a test strip that was read by the monitor. Participants who used cervical mucus monitoring were asked to check daily for low, high, or peak rated mucus whenever voiding and at the end of the day and to record the most fertile level of cervical mucus observed as in previous studies of this same method (36). Charts were only included if they had enough information to discern (both for the user and for the practitioner) the estimated fertile window.

### Pregnancy Rates

Users are notified by the online charting system of the possibility of a pregnancy when the post ovulatory phase of the charted menstrual cycle is greater than 19 days. When this happens, the charting system prompts the user to take a pregnancy test and to take an online a pregnancy evaluation. Once the evaluation is completed, professional nurses review it along with the charts and a determination is made if intercourse occurred during the fertile time as designated by the online charting system instructions.

Pregnancy rates were determined by using survival analysis (Kaplan–Meier) with the Statistical Package for Social Sciences (SPSS, version 21). Pregnancies were recorded as correct use when there was an indication of intercourse during the estimated fertile phase on “High” and “Peak” fertility days. Correct use pregnancy rates were calculated based on 100 women per 12 menstrual cycles of use and included only menstrual cycles that were determined to be correct use, i.e., intercourse during the fertile window on “High” and “Peak” days (see Figure 1). Correct use pregnancy rates of the total 256 women participants and subgroups of participants who used either the fertility monitor, cervical mucus, or both indicators of fertility were determined. Chi-square analysis was used to determine differences in the frequency of pregnancies among the subgroups of participants using the fertility monitor, cervical mucus observations, or both to estimate the fertile window of the menstrual cycle. Logistic regression, with pregnant or not as the dependent variable and predictive factors of age, education, number of living children, and length of time trying to achieve a pregnancy was run as an auxiliary equation for further information and understanding.

## Results

### Demographics

The 256 participants had a mean age of 29.22 (SD = 4.7; range 20–43), were married a mean of 3.7 years (SD = 4.1; range <1–19), and had a mean of 1.4 children (SD = 1.6; range 0–9). Most (80%) were college graduates, 93% Catholic, and 83% Euro-American. The mean number of months attempting pregnancy was 3.4 months (SD = 11.7: range 0–131 months).

### Total Pregnancy Rates

The total number of pregnancies for the 256 participants was 150 or 59% of the participants (Table 2). The cumulative pregnancy rates were 58 per 100 women at 3 cycles of use, 73 at 6 cycles of use, 75 at 9 cycles of use, and 78 at 12 cycles of use. However, carried out to 24 cycles of use the cumulative pregnancy rate is 86 per 100 users.

**Table 2:** Overall pregnancy rates (per 100 women) for all participants, for those who were trying to achieve pregnancy since the first cycle of use, and for those with at least one previous pregnancy.

Cycles of use	Overall (*N* = 256)	Trying from first cycle of use (*N* = 181)	At least one previous pregnancy (*N* = 153)
3	58	66	66
6	73	80	82
9	75	81	82
12	78	84	86
24	86	90	90

The total number of pregnancies for the 181 trying to achieve pregnancy from the first cycle of use onward was 127 or 70% out of 181 participants. The cumulative pregnancy rates were 66 per 100 users at 3 cycles of trying, 80 at 6 cycles of trying, 81 at 9 cycles, 84 at 12 cycles, and 90 at 24 cycles of trying.

The total number of pregnancies for the 153 trying to achieve pregnancy with at least 1 living child was 99 or 65%. The cumulative pregnancy rates were 66 per 100 users at 3 cycles of trying, 82 at 6 and 9 cycles of trying, 86 at 12 cycles, and 90 at 24 cycles of trying.

### Pregnancy Rates by Fertility Indicator

The correct use pregnancies for the participants by fertility indicator was 54 (67.5%) out of 80 using the fertility monitor to estimate the fertile window, 11 (44%) out of 25 using cervical mucus monitoring, and 91 (61%) out of the 148 using both indicators. Chi-square analysis showed a significance difference in frequency of pregnancies among the three sub groups *X* = 7.13, *p* = 0.028. There was a greater frequency in pregnancies between the fertility monitor and the mucus group *X* = 6.91, *p* = 0.009 but not between the mucus and mucus plus monitor participants, nor between the monitor and monitor plus mucus group. Survival analysis subgroup comparison also showed a significant difference in rates of pregnancy between the monitor and mucus groups (*X* = 4.63, *p* = 0.03) but not in the other groups.

The cumulative pregnancy rates by fertility indicator (Table 3) was 80 at 6 cycles of use, 83 at 12 cycles, and 100 per 100 women at 24 cycles of use with use of the fertility monitor; for cervical mucus, the rates were 48 at 6 months, and 72 at 12 cycles of use (not enough power for further analysis), and for both indicators, 69 at 6 cycles of use, 75 at 12 cycles of use, and 79 at 24 cycles of use.

**Table 3:** Correct use pregnancy rates (per 100 women) according to fertility indicator.

Cycles of use	Monitor	Mucus	Monitor + mucus
6	80	48	69
12	83	72	75
24	100	Not enough power to calculate	79

The total number of pregnancies by fertility indicator for the 181 trying to achieve for the first time in the online charting system was 43 (78%) out of 55 participants for the monitor; for cervical mucus, 9 (69%) pregnancies out of 13 participants, and for both indicators, 75 (66%) out of 113 participants. The cumulative pregnancy rates for the fertility monitor participants were 87, 99, and 100 for 6, 12, and 24 cycles of use per 100 users, for cervical mucus, 81 at 6 cycles of use (not enough data for further analysis), and for both indicators 76, 80, and 84 at 6, 12, and 24 cycles of use.

The total number of pregnancies by fertility indicator for the 153 trying to achieve pregnancy and have at least 1 living child was 39 (79%) for the 48 participants using the fertility monitor, 9 (64%) for the 14 using cervical mucus, and 51 (57%) out of the 90 using both indicators. The cumulative pregnancy rates were 93% at 12 cycles of use for the fertility monitor participants, and 84 at 12 cycles of use for both mucus and monitor. There were not enough data for those using mucus only.

### Intercourse outside the Fertile Window

Intercourse focused on “High” and “Peak” days on the CBFM had a pregnancy rate of 85 per 100 women over 12 months of use. When intercourse was outside this fertile window (on low days on the monitor), there was only 1 pregnancy per 100 women over 12 months of use.

### Logistic Regression

A logistic regression equation with “pregnancy or not” as the dependent variable, and age, number of years of schooling, number of living children, and time attempting to achieve a pregnancy was significant (*p* < 0.005) (Table 4). The significant Betas were number of school years (*p* = 0.033) with a 17% greater likelihood to achieve pregnancy with more school years, living children (*p* = 0.045) with having more children providing 27% greater likelihood to achieve pregnancy, and time to pregnancy (*p* = 0.018) with a 10% less likely chance of achieving a pregnancy with the greater time trying to achieve.

**Table 4:** Logistic regression analysis of the likelihood of achieving pregnancy.

Variable	*B*	SE	Wald	Df	Sig	Exp(*B*)
Age	−0.063	0.040	2.433	1	0.119	0.939
School years	0.159	0.074	4.558	1	0.033	1.172
Living children	0.237	0.118	4.014	1	0.045	1.267
Time attempting	−0.104	0.044	5.617	1	0.018	0.902
Constant	0.899	1.096	0.674	1	0.412	2.458

## Discussion

Although the overall pregnancy rates among the total participants by 12 cycles of use (78 pregnancies per 100 users) is less than the predicted 85 per 100 women that is provided by Trussell (14) for women who have unprotected intercourse over 12 months and the 92 per 100 women at 12 cycles of use that Gnoth et al. found with timed intercourse (31), nevertheless, by 24 cycles of use the overall pregnancy rate goes up to 86 per 100 women. Gnoth et al. (31) also found that if couples continue to have focused intercourse a good proportion who have not achieved in the first 12 months of trying will eventually get pregnant. Of interest is that participants who were trying to achieve for the first time as well as those with children, by 24 cycles of use with focused intercourse 90 per 100 were able to achieve a pregnancy. Both of these subgroups and the total participants’ pregnancy rates affirm the benefit of using focused intercourse for 24 cycles of use. Participants in this study may have lower fertility than the population at large since they were seeking additional resources to help conceive, so it is not surprising that this study’s pregnancy rates may be slightly lower than the general population. Since we did not screen specifically for infertility, these results could not be generalized to an infertile population; however, the fact that the sample was gathered from those seeking to achieve pregnancy, it could be applied to a primary care situation where women are seeking easily accessible tools to assist in achieving pregnancy.

The pregnancy rate for the participants that only used the CBFM had an increase in cumulative pregnancy rates from 83 at 12 cycles of use to 100 at 24 cycles of use. The pregnancy rate for the mucus plus monitor group at 24 cycles of use only reached 79 per 100 users. The group using only mucus as an indicator did not have enough power for the 24-month analysis. Overall, the frequency of pregnancy was greater for the monitor versus the mucus group by chi-square analysis. In this study, when the CBFM alone was used by women participants who were trying to achieve for the first time they had a 99 per 100 pregnancy rate at 12 months and 100 at 24 months, which again was better than the mucus and mucus plus monitor group, possibly because the fertility monitor indicator is a more objective indication of fertility. Previous studies with a hormonal fertility monitor compared with control groups also showed a higher pregnancy rate with use of the fertility monitor (19, 34). Even though the results of this study show the use of the CBFM alone seems to achieve the highest pregnancy rates, the use of other fertility indicators like mucus should not be excluded based on this study—couples who would like to use multiple indicators or even mucus alone should be given the support needed by their primary care providers in identifying their fertile window.

The results from the logistic regression analysis suggested that having a previous full-term pregnancy increased the likelihood of subsequently achieving pregnancy. It is also not surprising from this analysis that couples trying for more than one cycle of use had an increased pregnancy rate. The contribution of a higher level of education may imply that more educated women might have greater ability to seek fertility information and to follow instructions for achieving pregnancy.

The main weakness of this study is that there is no comparison with couples who use random and frequent intercourse to achieve pregnancy. Unfortunately, our results do not allow us to determine whether focused intercourse provides a shorter time to pregnancy than random, frequent intercourse. However, in a previous study (35), there was a higher pregnancy rates when couples focused intercourse on High and Peak days versus low days in the estimated fertile window. We found similar results in this study: A further weakness was that although the study was prospective and menstrual cycles and intercourse patterns were charted over time, there was no follow-up on those who discontinued charting. In addition, as in past studies with online self-recording, there is an under-reporting of intercourse as some participants feel this is too private or do not wish to show that they are not following instructions. Furthermore, the actual pregnancy rates are most likely higher than what has been reported in this study, as couples stop recording menstrual cycles and do not always inform the professionals managing the web site of their pregnancy.

The findings in this study are consistent with the Practice Committee of the American Society for Reproductive Medicine (1) stating that fertility monitors might be helpful for couples trying to achieve pregnancy to focus intercourse on the fertile window of the menstrual cycle. However, not all fertility monitors provide the same information. The CBFM provides direct measurements of the urinary metabolites of estrogen and LH. There is still a question as to whether focused intercourse with these fertility monitors or with cervical mucus monitoring is more effective than just frequent intercourse. A large randomized trial or a cohort comparison study among groups using frequent intercourse, hormonal monitoring, and/or cervical mucus monitoring would be beneficial.

## Conclusion

The use of focused intercourse and menstrual cycle charting with online systems and fertility monitoring apps is a simple cost-effective first step in helping couples to achieve pregnancy, in combination with other primary care interventions described in Table 1. Not only is fertility cycle charting helpful to identify the fertile window, it can also be used as an assessment tool to identify possible fertility problems such as short luteal phases, anovulatory menstrual cycles, long cycles with long follicular phases, unusual uterine bleeding patterns, and polycystic ovarian syndrome to name a few examples. It is well within the domain of primary care providers to begin providing interventions for couples to achieve pregnancy before any fertility investigations are needed, and without the need to pursue ARTs. As demonstrated in this study, many will become pregnant by 12 cycles of intercourse focused in the fertile window, and for those in the monitor group, 100 per 100 women were pregnant after 24 cycles of use.

## Ethics Statement

The study received human subject approval through the Marquette University Office of Research compliance (HR-1597).

## Author Contributions

The corresponding author collated the data; collection and analysis were provided by all three authors; writing portion was contributed by each author.

## Conflict of Interest Statement

The authors declare that the research was conducted in the absence of any commercial or financial relationships that could be construed as a potential conflict of interest.
